# An efficient enzyme-triggered controlled release system for colon-targeted oral delivery to combat dextran sodium sulfate (DSS)-induced colitis in mice

**DOI:** 10.1080/10717544.2021.1934189

**Published:** 2021-06-12

**Authors:** Shangyong Li, Mengfei Jin, Yanhong Wu, Samil Jung, Dandan Li, Ningning He, Myeong-sok Lee

**Affiliations:** a School of Basic Medicine, Qingdao Medical College, Qingdao University, Qingdao, China; b Molecular Cancer Biology Laboratory, Cellular Heterogeneity Research Center, Department of Biosystem, Sookmyung Women's University, Seoul, Korea

**Keywords:** Colon-targeted drug delivery, enzyme-triggered controlled release, ulcerative colitis, intestinal homeostasis, gut microbiota

## Abstract

Oral route colon-targeted drug delivery systems (CDDSs) are desirable for the treatment of ulcerative colitis (UC). However, CDDSs are challenging owing to the physiological and anatomical barriers associated with the gastrointestinal tract (GIT). In this study, we developed an effective enzyme-triggered controlled release system using curcumin–cyclodextrin (CD–Cur) inclusion complex as core and low molecular weight chitosan and unsaturated alginate resulting nanoparticles (CANPs) as shell. The formed CD–Cur–CANPs showed a narrow particle-size distribution and a compact structure. *In vitro* drug release determination indicated that CD–Cur–CANPs showed pH-sensitive and α-amylase-responsive release characteristics. Furthermore, *in vivo* experiments demonstrated that oral administration of CD–Cur–CANPs had an efficient therapeutic efficacy, strong colonic biodistribution and accumulation, rapid macrophage uptake, promoted colonic epithelial barrier integrity and modulated production of inflammatory cytokines, reshaped the gut microbiota in mice with dextran sodium sulfate (DSS)-induced colitis. Taken together, our synthetic CD–Cur–CANPs are a promising synergistic colon-targeted approach for UC treatment.

## Introduction

Ulcerative colitis (UC) is an idiopathic chronic inflammatory disease of the colonic mucosa, with adverse public health effects (Kobayashi et al., 2020). Oral drug delivery systems (ODDSs) are highly desirable for the treatment of UC as they improve therapeutic efficiency and reduces systemic toxicity (Han et al., 2019). Oral colon-targeted drug delivery is of great interest for UC therapy. However, due to physiological challenges, biochemical, and environmental barriers, it is difficult to target oral drugs to the colon (Wang et al., 2016).

Thus far, several natural, synthetic, and semi-synthetic polymers have been used to overcome the strong physiological variations in the upper gastrointestinal tract (GIT) and focus drug release in the colon (Bansal et al., 2014; Arévalo-Pérez et al., 2020). Among them, chitosan–alginate nanoparticles (CANPs) have attracted increasing interest as colon-targeted ODDSs. Chitosan is a cationic polymer derived from chitin (Rivera et al., 2015), while, sodium alginate is a linear hetero-polyuronic acid polymer extracted from seaweed (Tønnesen & Karlsen, 2002; Li et al., 2020). Due to their biodegradability, biocompatibility, adhesion, safety, and gel properties, both chitosan and alginate are widely used in drug delivery (Azevedo et al., 2014; Maity et al., 2017; Sorasitthiyanukarn et al., 2018). Moreover, the strong electrostatic interaction between the carboxyl group of alginates and the amino group of chitosan leads to shrinkage and gel formation at low pH (Thai et al., 2020), which enhance their protection from low gastric pH, and to provide release in intestinal conditions (Mukhopadhyay et al., 2015).

Recently, advanced stimuli-responsive controlled release systems have received increasing attention for drug delivery (Descalzo et al., 2006), which can regulate the release of entrapped drug molecules by specific external stimuli such as light (Schroeder et al., 2012), temperature (Ruiz et al., 2020), magnetic fields (Tasciotti, 2018) or by internal enzymes (Zhang et al., 2014). Gut microbiota are microorganisms coexist peacefully with the host in their gut and secret several carbohydrate-active and reductive metabolizing enzymes (Peng et al., 2021). Intestinal enzymes are unique stimuli that are increasingly being used as triggers in controlled release systems due to their high substrate specificity (Mura et al., 2013). Cyclodextrin (CD) are natural cyclic oligosaccharides linked by α-1,4-glucoside bonds with cone-like conformation, which express an external hydrophilic surface and a relatively hydrophobic inner cavity. Meanwhile, CD could open the ring under the action of colonic microorganism fermentation and enzymatic hydrolysis, then the ester bond is hydrolyzed and the drug is released in colon (Park et al., 2009). Curcumin (Cur) is a natural yellow polyphenol occurring in turmeric roots, with proven pharmacological activities, including anti-inflammatory, anti-bacterial, anti-viral, anti-tumor, anti-ulcer, immunomodulatory, and neuroprotective effects (Liu et al., 2016; Nelson et al., 2017; Di Meo et al., 2019). Current studies have shown that Cur is therapeutically effective against UC (Grammatikopoulou et al., 2018; Sadeghi et al., 2020). However, the poor water solubility and instability of Cur lead to poor oral absorption and low bioavailability in the GIT, which limits its application in oral therapy (Manju & Sreenivasan, 2011).

In this study, we developed a novel enzyme-triggered controlled release system for colon-targeted drug delivery. The β-CD was selected as the carrier of curcumin (Cur), and Cur was utilized as a model drug for convenient detection. The core–shell NPs were successfully prepared using CD–Cur inclusion complex as core and CANPs as shell. The formed CD–Cur–CANPs showed narrow particle-size distribution and a compact structure. *In vitro* drug release determination indicated that CD–Cur–CANPs showed pH-sensitive and α-amylase-responsive release characteristics. Furthermore, *in vivo* experiments demonstrated that oral administration of CD–Cur–CANPs had an efficient therapeutic, strong colonic retention, promoted colonic epithelial barrier integrity, and reshape of gut microbiota in mice with dextran sodium sulfate (DSS)-induced colitis.

## Material and methods

### Materials and supplies

The chitosan (*M*_w_: 140–190 kDa, degree of deacetylation: >85%, the viscosity: 200–400 mPa s) was purchased from Aladdin (Shanghai, China). High-viscosity sodium alginate (*M*_w_: 20–50 kDa) was purchased from Bright Moon Seaweed Group (Qingdao, China). α-Amylase from *Aspergillus oryzae* was purchased from Aladdin (Shanghai, China). Dextran sodium sulfate (DSS) was supplied by MP Biomedicals (Irvine, CA). Cur and β-CD were purchased from Solarbio (Beijing, China). Male C57BL/6 mice (8-week old, 18–22 g) were provided by Pengyue Laboratory Animal Technology Co., Ltd. (Jinan, China) and preserved in an aseptic environment. Mouse interleukin-6 (IL-6), tumor necrosis factor-α (TNF-α), and interleukin-1β (IL-1β) ELISA kits were purchased from Nanjing Jiancheng Bioengineering Institute (Nanjing, China). Rhodamine B isothiocyante (RBITC) and fluorescein isothiocyanate (FITC) were purchased from Macklin (Shanghai, China).

### Preparation and analysis of low molecular weight chitosan and unsaturated alginate

The purchased high molecular weight chitosan was dissolved in 1 M aqueous acetic acid (HAc) to a concentration of 1% (w/v) and chitosanase CsnM (20 U/mL) was added to colloidal chitosan at 20 °C for 10 min in a shaky condition. Low molecular weight unsaturated alginate was prepared by oligoalginate lyase OalC6 in our lab (Li et al., 2018). OalC6 (1 U) was added to 1 mL of high-viscosity sodium alginate polymer (5 mg/mL in 20 mM phosphate buffer, pH 7.0) and incubated at 40 °C for 60 min in a shaky condition. Then, these degrading products were heat-treated and dialyzed by a 1000 Da dialysis bag to remove smaller chitosan oligosaccharides (COS) or alginate oligosaccharides (AOS). The average molecular weight of prepared low molecular weight chitosan (8.76 kDa) and unsaturated alginate (7.73 kDa) were analyzed using viscosity methods (Li et al., 2019).

### Cyclodextrin–curcumin (CD–Cur) preparation

The molar ratio of CD to Cur was 1:1 (v/v). Briefly, 647 mg of CD was dissolved in 120 mL water, and 147 mg curcumin was dissolved in 2 mL acetone. The oil phase was added to the aqueous solution and centrifuged at 400 rpmin. Uncovered acetone was volatilized by stirring for 24 h in the dark, and centrifuged at 1000 rpm for 5 min. The supernatant was collected and lyophilized to recover water-soluble CD–Cur.

### Preparation and determination of nanoparticles

The CD–Cur–CANPs were prepared by ion gel method. Briefly, 0.0315% (w/v) sodium alginate solution and 0.07% (w/v) chitosan were dissolved in water and 1% acetic acid solution, respectively, and stirred overnight. Then, 1 mL of water-soluble CD–Cur and 23.5 mL of prepared low molecular unsaturated alginate solution were mixed and evenly dispersed, followed by the drop-wise addition of 0.2% (w/v) 1.5 mL calcium chloride solution at a speed of 0.1 mL/min, and stirred for 30 min to form a pre-gel. Finally, 2 mL low molecular weight chitosan solution was added at a rate of 0.1 mL/min and stood for 30 min to form an evenly dispersed solution. After centrifugation at 12,000 rpm for 30 min, the supernatant was discarded and washed three times with ultra-pure water. The powder was obtained by vacuum freeze-drying. The particle size and the zeta potential of the synthetic CD–Cur–CANPs were measured *via* dynamic light scattering (DLS) with Zetasizer Nano ZS (Malvern Instrument, Malvern, UK). Morphological analysis was performed using transmission electron microscopy (TEM) in open field mode. Fourier transform infrared (FTIR) spectroscopy was used to analyze the samples with a FTIR spectrometer in the range of 4000–400 cm^−1^. Before the measurement, the samples were dried under vacuum until a constant weight was reached. The dried samples were pressed into the powder, mixed with the KBr powder, and then pressed into a shape for FTIR spectroscopy to measure the wavelength.

### Encapsulation efficiency (EE) and drug release

The drug EE and loading efficiency (LE) were determined using a direct method by dispersing 5 mg synthetic CD–Cur–CANPs in 10 mL anhydrous ethanol and oscillated at a constant temperature of 37 °C for 24 h to obtain completely swollen NPs. Then, the solution was cleaned ultrasonically for 30 min and centrifuged at 12,000 rpm for 30 min. The concentration of Cur in the supernatant was measured using an ultraviolet spectrophotometer at 425 nm according to the pre-determined standard curve. Finally, the EE and LE were calculated using the following formula:

$$\mathrm{EE}\%=\frac{\text{Weight of the drug in NPs }(\mathrm{mg})}{\text{Weight of the total drug }(\mathrm{mg})}\;\times \;100\%$$

$$\mathrm{LE}\%=\frac{\text{Weight of the drug in NPs }(\mathrm{mg})}{\text{Weight of the total NPs }(\mathrm{mg})}\;\times \;100\%$$

A dialysis bag was used to determine the pH sensitivity and enzyme responsive release characteristics of the formed nanoparticles. First, 5 mg formed Cur–CD–CANPs were dispersed in 10 mL water and transferred to a dialysis bag. The pH sensitivity of formed Cur–CD–CANPs were performed at 100 mL different solution buffer (pHs 1.2, 6.8, and 7.4) and shaken (100 rpm) at 37 °C for 12 h. *In vitro* release profiles of Cur from in the presence and absence of α-amylase (10 IU/mL) were carried out for 12 h in 100 mL PBS (pH 7.4) to determine the enzyme responsive release characteristics. Simultaneously, samples were removed at different times and the concentration of free Cur was measured by fluorescence spectroscopy (425 nm).

A basket rotation method was used to simulate the process of oral drugs entering the human gastrointestinal tract. First, the NPs were exposed to a simulated gastric acid environment (pH 1.2) for 2 h, followed by transferring to a pH 6.8 buffer system to simulate the small intestine environment for another 2 h. Finally, they were transferred to pH 7.4 to simulate the colon environment for 8 h. PBS (pH 7.4) with α-amylase (10 IU/mL) were performed under the same conditions. Meanwhile, 2 mL aliquots of sample solution were removed at different times to detect the free Cur content, and mixed with an equal amount of fresh slow-release medium.

The cumulative drug release rate of the compound particles at time T was calculated as follows:

$$\text{Drug cumulative release}=\frac{\text{Cumulative drug release }(\mathrm{mg})}{\text{The total amount of particle loading }(\mathrm{mg})}\times 100\%$$

### Fluorescence-labeling of chitosan

The synthesis of marked low molecular weight chitosan with RBITC or FITC was based on the reaction between the isothiocyanate group of RBITC/FITC and the first-order amino group of chitosan (Cheng et al., 2018). Briefly, 0.5 g prepared chitosan was dissolved in 100 mL of 2% (v/v) acetic acid solution by electromagnetic stirring for 30 min. Then, 1 mol/L of NaOH was used to adjust the pH to 7.5. Under stirring, 1 mL of DMSO solution containing RBITC or FITC (1 mg) was added to the chitosan solution. After reacting in the dark at 40 °C for 1 h, the reaction was carried out at room temperature overnight. The solution was then centrifuged at 4000 rpm for 10 min, and washed with distilled water repeatedly to remove free RBITC or FTIC, until a clear supernatant was obtained. The RBITC-labeled or FITC-labeled chitosan was used to prepare Fluorescence-labeled CD–Cur–CANPs according to the above protocol.

### Cellular uptake of nanoparticles on RAW264.7 macrophages

Mouse monocyte macrophages (RAW264.7) were purchased from the China Center for Type Culture Collection (Wuhan, China) and maintained in accordance with supplier's instructions. RAW264.7 cells were seeded onto glass coverslips in 24-well plates (4 × 10^4^ cells per well) and incubated at 37 °C in 5% CO_2_ for 4 h. Then, the medium was replaced with a fresh medium containing 100 µg/mL FITC-labeled CD–Cur–CANPs. After being treated in indicated time, the medium was removed and washed with PBS twice. Cells were fixed in 4% paraformaldehyde for 20 min and washed three times with PBS. Then, cells were incubated in 4′,6-diamidino-2-phenylindole dilactate (DAPI) labeling solution (0.5 µg/mL in PBS) for 5 min at room temperature and without light. Finally, the images were acquired using a DS-Mv digital camera (Nikon, Tokyo, Japan) mounted on a BX53 microscope (Olympus, Tokyo, Japan). The light intensity and exposure time of each dye spot were set as the same condition. All experiments were done in triplicate.

### Animal model and treatments

Male C57BL/6 mice (8 weeks old, 20–22 g) were maintained in a temperature and humidity-controlled facility (25 ± 2 °C, 50 ± 5% relative humidity) under a 12 h light/dark cycle with unrestricted access to a diet of chow and water. All the animal experiments were approved by the Ethics Committee of Medical College of Qingdao University. After acclimation for 1 week, mice were divided into five groups randomly (*n* = 6 per group) as follows: (1) Ctrl: negative control group and mice received regular drinking water; (2) DSS model group; (3) CD–Cur-treated DSS group; (4) CD–Cur–CANPs-treated DSS group; and (5) RBITC-labeled CD–Cur–CANPs-treated DSS group. The mice in the drug-treated groups received an equal dose of Cur (50 mg/kg) in the form of suspension by oral gavage for 7 days. Mice were given 3% DSS (molecular weight 36–50 kDa) instead of drinking water for 7 consecutive days to induce colitis. The state of mice in groups 1–4 was carefully recorded and monitored daily for signs of disease (weight loss, stool consistency, and rectal bleeding). The disease activity index (DAI) was calculated according to the percentage of daily weight loss, stool consistency and average score of fecal blood/occult blood, as shown in Table S1. At the end of the experiment, feces were collected from groups 1–4 and stored at −80 °C for further analysis. Colon and spleen tissues were quickly stripped and weighed. The tissues were photographed and then also stored at −80 °C for further analysis.

### *In vivo* biodistribution of nanoparticles

The mice in group 5 above were used to determine the tissue-targeting effect of synthetic NPs in colitis. RBITC-labeled CD–Cur–CANPs (100 μL) were administered on day 7 after the colitis model was established. To avoid background interference due to food in the GIT, the feeding of the mice was restricted 6 h before imaging. After 6 h and 12 h, the tissues and living bodies of the mice were imaged using the small animal *in vivo* 3D imaging system (PerkinElmer Life Sciences, Boston, MA). Furthermore, the fluorescent colon tissue at 6 h were selected to prepare frozen slices and microscopically magnified to depict the targeting uptake and distribution of the RBITC-labeled CD–Cur–CANPs.

### Hematoxylin and eosin (H&E) staining and ELISA assay

H&E staining was performed by Sevicepio (Wuhan, China). The colon segment was fixed in 4% paraformaldehyde solution for 24 h, dehydrated, infiltrated and embedded in paraffin. The 4-μm-thick sections were obtained with the slicer and placed on the slide. Sections were stained with H&E. Images were obtained using a microscope at 200× and 400× magnification.

The colon tissues were homogenized in ice-cold PBS (pH 7.4). The homogenates were centrifuged at 14,000 rpm for 5 min at 4 °C, and the supernatants were collected. Concentrations of IL-1β, IL-6, and TNF-α were measured according to the ELISA kit instructions. The results were expressed as pg of cytokines per mg of total protein in the colon homogenate.

### Real-time quantitative PCR

Total RNA was isolated from tissues using a mirVana miRNA Isolation Kit (Ambion, Carlsbad, CA) in accordance with the manufacturer's instructions. The study primers were synthesized by Sangong Biotech (Shanghai, China) (Table S2). Gene expression levels were normalized to GAPDH, followed by the relative quantification of gene expression using the 2^−ΔΔCt^ method. All real-time PCR reactions were performed in triplicate, as previously described (Song et al., 2018; Li et al., 2019).

### Western blot

Total protein in the colon tissue was extracted using RIPA tissue lysate, and protein concentration was determined using a bicinchoninic acid (BCA) protein quantification kit. Western blotting analysis was performed as previously described (Song et al., 2018; Li et al., 2019).

### Fecal microbial species analysis

The qPCR was used to analyze the microbial composition of each sample. Total DNA of fecal microbial samples was extracted using a TIANamp Stool DNA Kit (Tiangen Biotechnology Co., Ltd., Beijing, China). The concentration of DNA samples was measured using a NanoDrop spectrophotometer (ThermoFisher, Pittsburgh, PA). The microbial groups were quantified via RT-PCR (Applied Biosystems 7500 Real-Time PCR System, ABI Co., Ltd., Foster City, CA) according to the instructions of the Power SYBR® Green PCR Master Mix Kit (ABI Co., Ltd., Foster City, CA). The reaction was initiated by activation at 95 °C for 5 min, followed by 40 target cDNA amplification cycles (denaturation at 95 °C for 15 s and extension at 60 °C for 35 s). Standard DNA templates were used to quantify target DNA copy numbers. Briefly, a series of 10-fold gradient dilutions of the standard product were used, and at least six non-zero standard concentrations were applied for each assay. The primers used were listed as Table S3. The concentration was expressed as log_10_ copy number.

### Statistical analysis

We performed statistical analysis using GraphPad Prism software (GraphPad Software, La Jolla, CA). The results were expressed as mean ± SEM. To determine the statistical significance between the two groups, we performed Student’s *t*-test to calculate the associated *p*-values. Statistical significance among multiple groups was calculated by one-way analysis of variance (ANOVA).

## Results

### Preparation and characterization of nanoparticles

**Figure 1.**
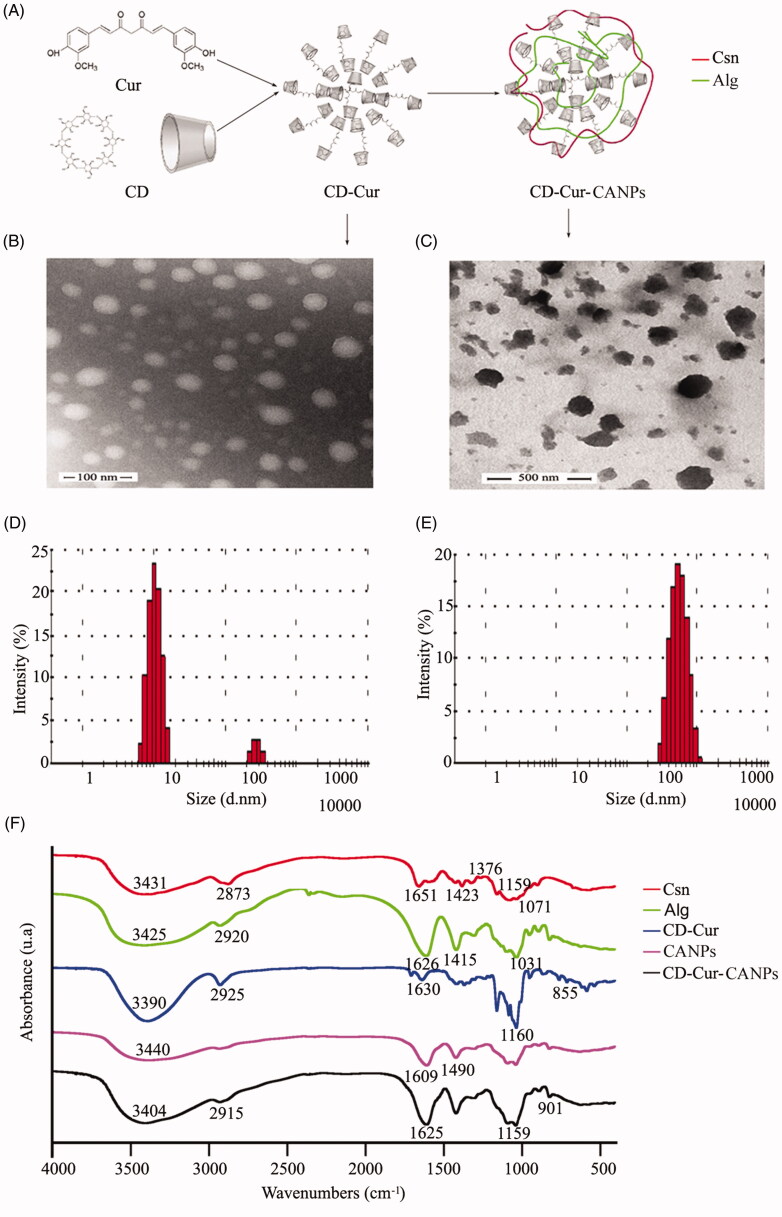
Preparation and characterization of nanoparticles. (A) The schematic diagram of prepared CD–Cur–CANPs. The TEM analysis of CD–Cur complex (B) and CD–Cur–CANPs (C); the size distribution of CD–Cur complex (D) and CD–Cur–CANPs (E); (F) FTIR spectra of nanoparticles.

The CD–Cur–CANPs were prepared by ion gel method using low molecular weight chitosan (8.76 kDa) and unsaturated alginate (7.73 kDa) as polymeric shells. Then, water-soluble CD–Cur inclusion complex as core encapsulated into CANPs shell (Figure 1(A)). The strong electrostatic interaction between the carboxyl group of alginates and the amino group of chitosan leads to shrinkage and gel formation at low pH (Thai et al., 2020), which increases its pH sensitivity and protects the drugs from GIT and the aggressive gastric environment (Mukhopadhyay et al., 2015). As presented in Figure 1(B), the CD–Cur complex tended to self-aggregate in the water solution, which showed simple micelles with a particle size of about 30–50 nm. The size of formed CD–Cur–CANPs is about 100–200 nm, which facilitated the adhesion and mobility of microspheres *in vivo* (Figure 1(C)). Dynamic light scattering (DLS) image shows that the average particle size of the formed CD–Cur is 84.04 nm (Figure 1(D)), and the average particle size of CD–Cur–CANPs is 462.1 nm (Figure 1(E)). It should be noted that the particle size of TEM was smaller than that of DLS, which may be due to the drying process of TEM sample treatment, and the particle size measured in solution may be larger than that of TEM in the drying state. The polydispersity index (PDI) was 0.227 ± 0.17, which indicated a monodisperse size distribution. The zeta potential of the synthetic CD–Cur–CANPs was −19 mV, suggesting good stability of the formed NPs.

FTIR spectroscopy was used to evaluate the formation of the CD–Cur–CANPs (Figure 1(F)). The solid samples including low molecular weight chitosan and unsaturated alginate, CD–Cur complex, synthetic CANPs, and CD–Cur–CANPs were prepared for FTIR analysis. Compared with the similar peaks in the FTIR spectra of chitosan and alginate, the peaks corresponding to the NH_2_, C–O, and OH groups in the FTIR spectra of NPs also shifted significantly, which showed strong interaction between chitosan and alginate. By comparing the spectra of CANPs without drugs and drug-loaded CD–Cur–CANPs, it was observed that most of the characteristic absorption bands of CD–Cur were accompanied by a certain degree of change. These changes indicate that CD–Cur is successfully encapsulated in CANPs.

### EE, drug loading, and release capacity

**Figure 2.**
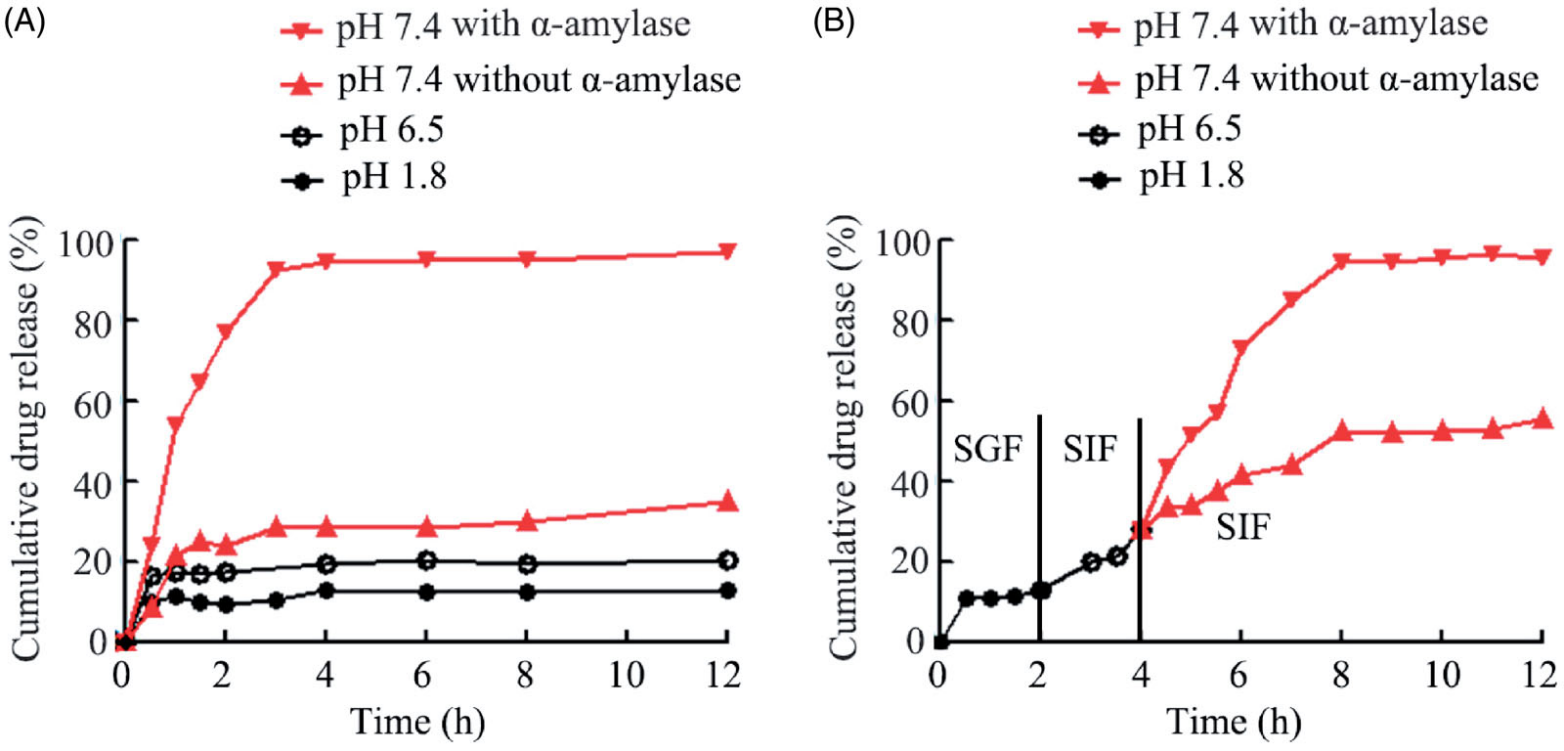
*In vitro* pH-dependent and enzyme responsive drug-release profiles of CD–Cur–CANPs. (A) Permeation rates of Cur in three different pH media (pHs 1.2, 6.8, and 7.4 with or without α-amylase, respectively). (B) Permeation rate of Curin gradually pH-changing medium (with or without α-amylase at pH 7.4) for 12 h.

The EE and drug loading (LC) of Cur–CD–CANPs were calculated indirectly by measuring the residual drug in the solution. The EE and LC of generated Cur–CD–CANPs were 88.89% and 3.49, respectively. The dialysis bag method was used to determine the permeation rate of Cur in three different pH media (pHs 1.2, 6.8, and 7.4, respectively). As shown in Figure 2(A), Cur has the maximum rate at pH 7.4. As pH decreases, the permeability decreases. Because of the strong electrostatic interaction between the carboxyl group of alginates and the amino group of chitosan leads to shrinkage and gel formation at low pH environment, Cur–CD–CANPs released only 15% of Cur at pH 1.2 within 12 h. These properties protect Cur from GIT and the aggressive gastric environment. To further confirm the protection of CANPs in the GIT, Cur–CD–CANPs were incubated in a medium with gradually changing pH to simulate the drug passage through the stomach (pH 1.2), small intestine (pH 6.8) and colon (pH 7.4). Only 12% and 27% of the Cur was released in the first 4 h at pH levels of 1.2 and 6.8 (Figure 2(B)), which represent the pH values of the stomach and the upper part of the small intestine, respectively.

The *in vitro* profiles of Cur release from Cur–CD–CANPs were studied in the presence and absence of α-amylase in the simulated ileum environment (pH 7.4). As shown in Figure 2, the release profiles indicated that the released Cur from CD–Cur was significantly higher in the presence of α-amylase compared to the absence of α-amylase. Cur was rapidly released from Cur–CD–CANPs with 10 IU of α-amylase and reached 90% within 4 h. This drastic release in the presence of α-amylase was owing to the β-CD enzymatic degradation and making chain scission in CD and drug release. These results indicate the formed NPs with pH sensitivity and enzyme responsive release characteristics.

### *In vivo* biodistribution and accumulation of nanoparticles in colitis mice

**Figure 3.**
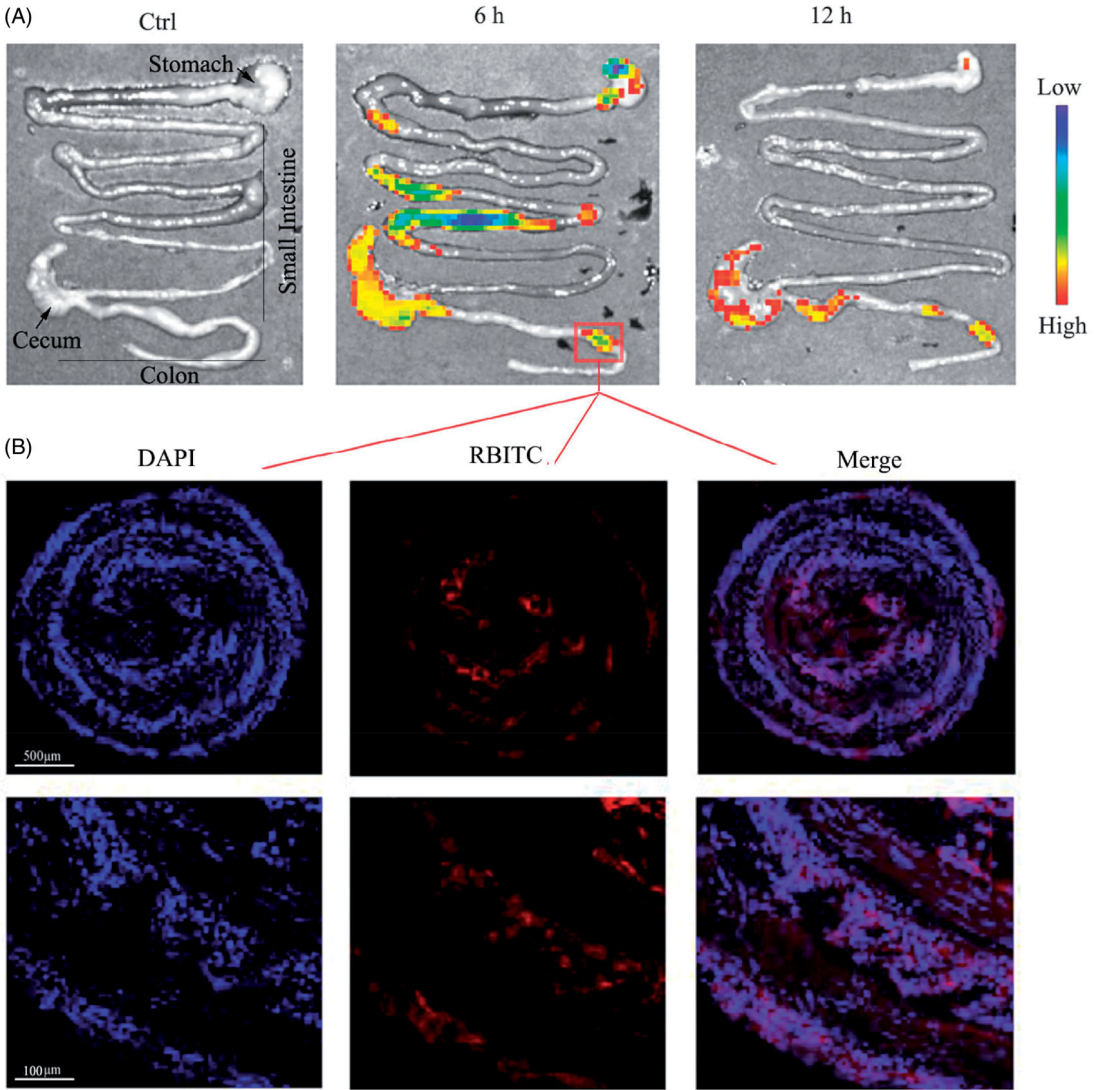
Biodistribution and accumulation of CD–Cur–CANPs in colitis mice. (A) The *ex vivo* inflorescence images were obtained to show the biodistribution of RBITC-embedded CD–Cur–CANPs after 6 h and 12 h administration. (B) Fluorescence microscopy images of colonic tissue slices at 6 h. Blue, DAPI for nuclear staining; red, RBITC-labeled nanoparticles.

To investigate the *in vivo* bio-distribution of CD–Cur–CANPs, we gavaged RBITC-embedded CD–Cur–CANPs in DSS-induced colitis mice, and analyzed the time-dependent passage and the *in vivo* targeting efficacy of this drug formulation using a small animal *in vivo* 3D imaging system. After oral administration of RBITC–CD–Cur–CANPs, the fluorescence intensities of whole mice were quantitatively analyzed at 6 and 12 h (Figure S1). The fluorescence signals after oral administration of synthetic NPs were primarily observed in the GIT, with faint signals in other internal organs. Furthermore, the percentage of RBITC dose in major GIT segments (i.e. stomach, small intestine, and colon including cecum) was assayed 6 and 12 h post-administration of NPs. *Ex vivo* RBITC imaging revealed that the signals accumulated throughout the digestive tract within 6 h. After 12 h of nanoparticle administration, the fluorescence intensities in the stomach and small intestine of UC mice decreased. Concurrently, strong signals were primarily observed in the colon at 12 h, indicating that the synthetic CD–Cur–CANPs exhibit strong colon-targeting biodistribution (Figure 3(A)).

Furthermore, the fluorescent colonic tissue slices were microscopically magnified to depict the targeting uptake and accumulation of the RBITC-labeled NPs. As shown in Figure 3(B), the colonic tissues of the colitis mice administered with CD–Cur–CANPs showed strong fluorescence signals (red), which indicated that CD–Cur–CANPs highly accumulated in the colonic tissues. Meanwhile, at 6 h post-administration of mice, CD–Cur–CANPs can pass through the intestinal barrier and cellular uptake through intrinsic action, indicating that the synthesized CD–Cur–CANPs exhibit strong colonic retention ability in colitis mice.

**Figure 4.**
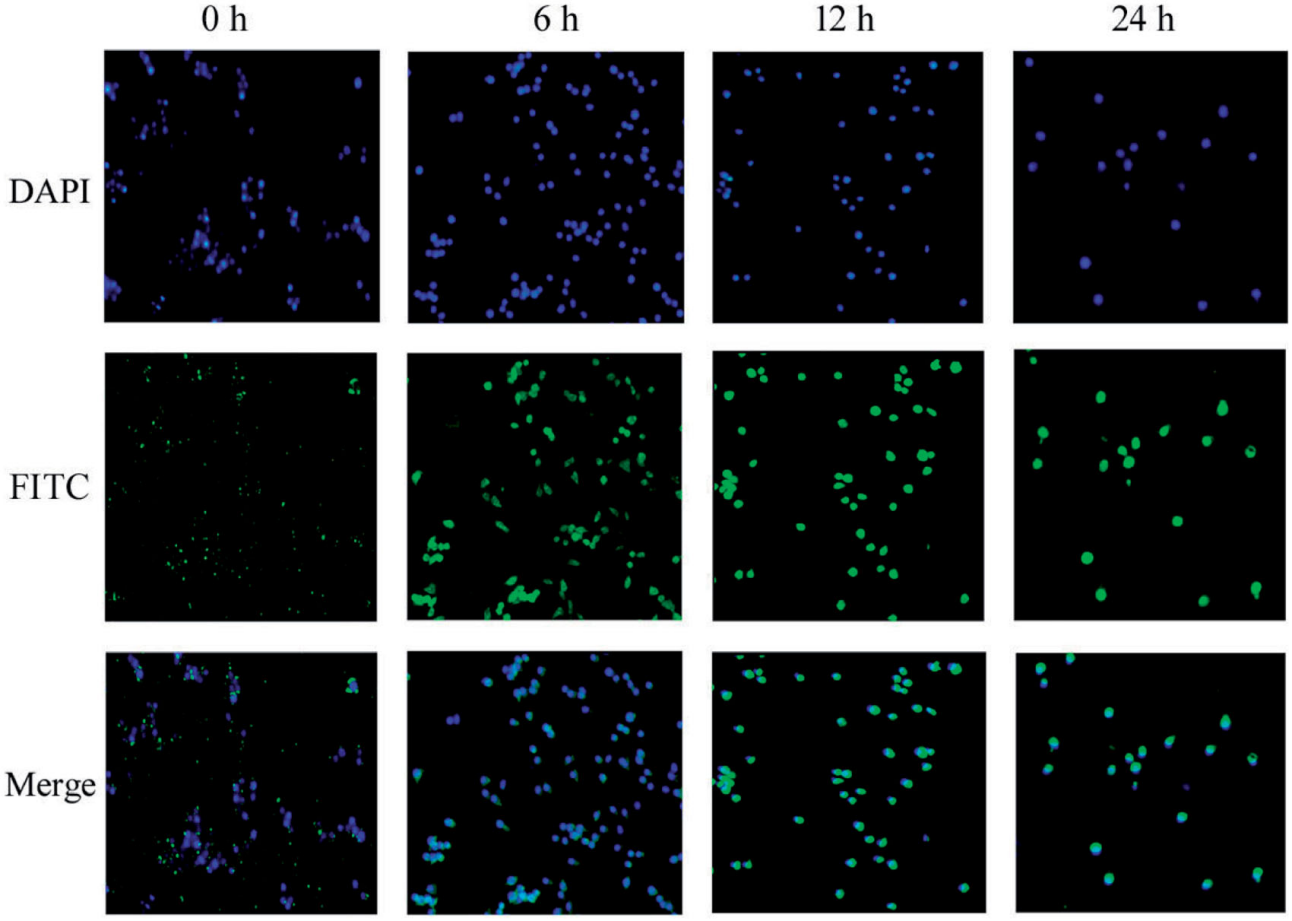
Fluorescence microscope of cellular uptake of nanoparticles on RAW264.7 cells. Blue, DAPI for nuclear staining; green, FITC-labeled nanoparticles.

To determine whether the formed CD–Cur–CANPs could be absorbed at the cellular level, CANPs was labeled with FITC dye (green fluorescence) and incubated with RAW264.7 cells *in vitro*. As shown in Figure 4, CANPs aggregated on the membrane of RAW264.7 cell at 0 h. After incubation for 6 h, FITC-labeled CANPs began to be incorporated into RAW264.7 macrophages and a small amount of green fluorescent substance could be observed in the cytoplasm. After incubation for 12 h and 24 h, almost all CANPs entered into RAW264.7 cells. These results indicate that CANPs be easily cellular uptake by RAW264.7 cells through a rapid and effective intrinsic action.

### *In vivo* therapeutic efficacy of CD–Cur–CANPs in DSS-induced colitis

**Figure 5.**
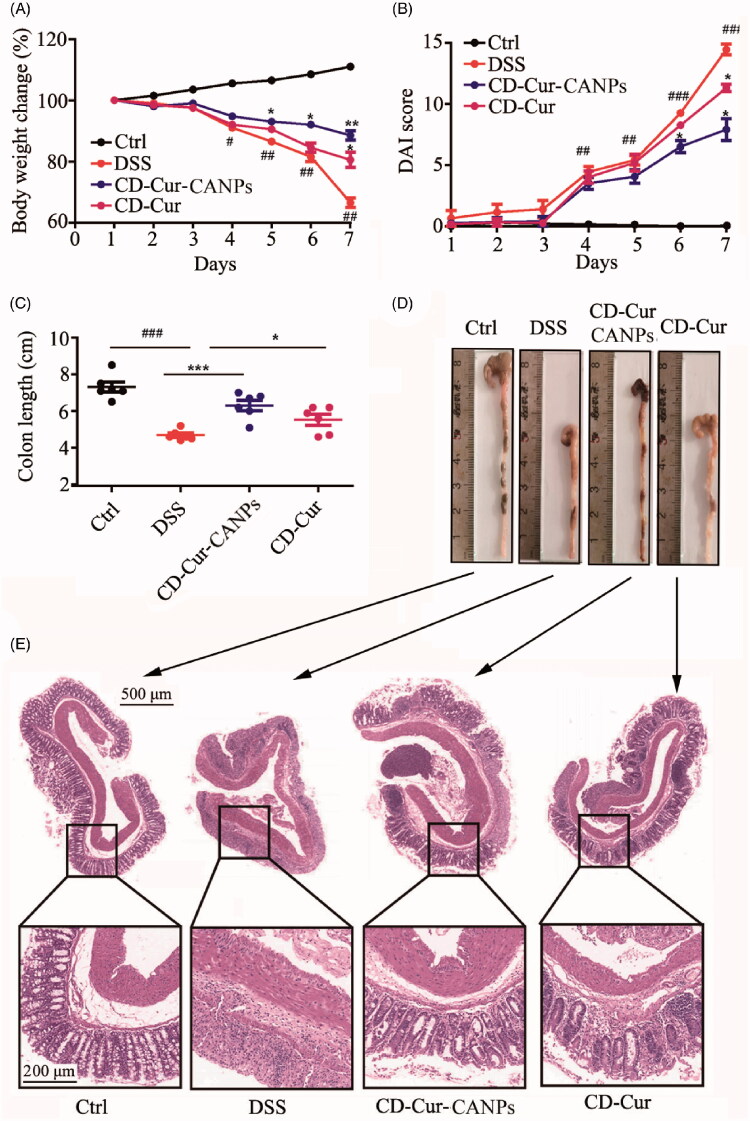
CD–Cur–CANPs ameliorated DSS-induced colitis. (A) Change in body weight during experiment. (B) Change in DAI score of each group. (C and D) Change in colon length of each group. (E) Effect of CD–Cur–CANPs on colon pathological changes of DSS-induced colitis mice. Representative histological photos for each group. Scale bars, 500 μm (original magnification, 50×) and 100 μm (original magnification, 200×). Compared to control group, ^#^*p <* 0.05, ^##^*p <* 0.01, ^###^*p <* 0.001. Compare to the DSS group, *p < .05, **p < .01, ***p < .001, na: no significance.

The DSS-induced colitis mouse model is easy to develop and repeat, and the symptoms are similar to human UC (e.g. weight loss, colon ulcers and bloody stools), and, thus, it has been widely used to evaluate new drugs or carriers for the treatment of UC (Perše & Cerar, 2012). In this study. DSS-induced acute colitis was established to evaluate the therapeutic effect of CD–Cur–CANPs on colitis. The colitis induction and treatment protocol is shown in Figure S2(A). As shown in Figure 5(A), mice treated with DSS showed significant loss of body weight from day 4, whereas the CD–Cur–CANPs significantly prevented the weight loss from day 5 (*p <* .05). Meanwhile, the severity of the disease was scored with DAI (Figure 5(B)). The DAI score of the colitis model group was significantly increased compared with CD–Cur–CANPs treatment group from day 6 (*p <* .05). On day 7, the DAI scores of both CD–Cur–CANPs and CD–Cur groups decreased significantly (*p <* .01). Administration of DSS induced severe inflammation in that DSS treatment significantly reduced the colon length (*p <* .001), whereas CD–Cur–CANPs treatment significantly reversed the reduction of colon length (*p <* .01, Figure 5(C)). DSS induction also significantly increased the weight of spleen (*p <* .05), and treatment with CD–Cur–CANPs significantly reversed the increase in spleen weight (*p <* .05, Figure S2(B)). These results suggest that treatment with CD–Cur–CANPs alleviated symptoms of colitis induced by DSS, with potential therapeutic application in colitis mice.

To further explore the inhibitory effect of CD–Cur–CANPs against histological damage of colon tissue in the mouse model of DSS-induced colitis, the severity of colonic damage and inflammation was analyzed using H&E staining (Figure 5(E)). As expected, the administration of DSS induced serious damage and inflammation in colon tissue, consistent with the DAI scores and colon morphology (Figure 5(B,C)). Compared with the control group, the DSS group showed severe erosion of colonic mucosa, destruction of almost all crypts, rapid decrease of goblet cells, inflammatory cell infiltration in lamina propria, glandular disorders and severe ulcers. CD–Cur treatment significantly reduced these symptoms of colitis, but the therapeutic effect was not comparable to that of CD–Cur–CANPs. The CD–Cur–CANPs group showed no apparent erosion of the colonic tissue, relatively intact crypts, neatly organized glands, complete goblet cells, and colon tissue morphology similar to that of the control group (Figure 5(E)). These results indicated that treatment with CD–Cur–CANPs decreased the inflammatory response of colon tissue in DSS-induced colitis mice model.

### Effect of CD–Cur–CANPs on intestinal homeostasis and gut microbiota

**Figure 6.**
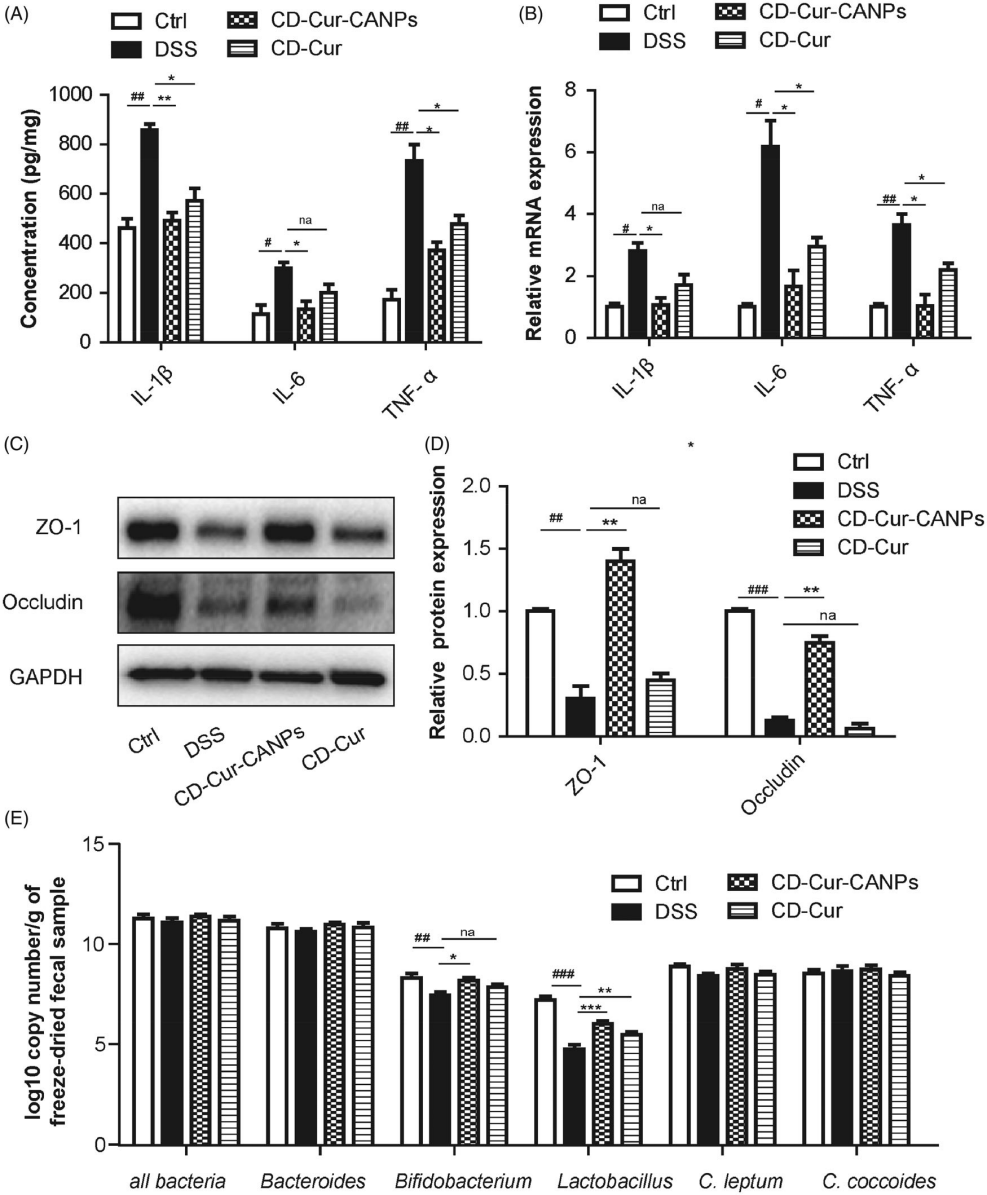
Effects of CD–Cur–CANPs on the inflammation, intestinal tight junctions and colonic microbiota composition in mice with colitis. Levels of inflammatory cytokines (IL-1β, IL-6, and TNF-α) in colon were determined by ELISA kits (A) and qPCR (B). The protein expression of ZO-1 and occluding were assessed using western blot (C) and quantitative analyzed (D). GAPDH was used to normalize the protein level and the relative ratio to control group. The relative abundance of microbiota species was determined by qPCR (E). Compared to the control group, ^#^*p <* .05, ^##^*p <* .01, ^###^*p <* .001. Compared to the DSS group, *p < .05, **p < .01; na: no significance.

Intestinal mucosal integrity could be significantly altered during active colitis aggravating the colon inflammation. To validate the anti-inflammatory effects of CD–Cur–CANPs, the protein concentration and gene expression levels of three important pro-inflammatory cytokines (IL-1β, IL-6, and TNF-α) associated with UC pathogenesis were detected (Figure 6(A,B)). The level of IL-1β, IL-6, and TNF-α in the colon samples were found to be increased in response to the induction of colitis in mice, whereas, significantly reduced (*p* < .01) after CD–Cur–CANPs treatment. These results suggest that CD–Cur–CANPs can effectively reduce inflammation in colon tissues of mice with DSS-induced colitis.

To further elucidate the role of CD–Cur–CANPs in modulating the intestinal tight junction, two major tight junction proteins (ZO-1 and occludin) were analyzed in colon tissues *via* western blot analysis (Figure 6(C,D)). DSS treatment significantly decreased the protein expression of ZO-1 (*p <* .01) and occludin (*p <* .001). Meanwhile, the expression of ZO-1 (*p <* .01) and occludin (*p <* .01), which was reduced by DSS treatment was significantly reversed by treatment with CD–Cur–CANPs. CD–Cur improved the protein expression of ZO-1 and occludin in DSS-induced colitis partially, but not significantly. The results suggest that treatment with CD–Cur–CANPs improved the integrity of the intestinal barrier in mice with DSS-induced colitis. These results indicated that CD–Cur–CANPs reduced the inflammatory response and improved the intestinal integrity of mice.

The protective effects of CD–Cur–CANPs against intestinal dysbacteriosis were quantitative analyzed by the change of gut microbial composition in the collected fecal pellet samples. The total levels of intestinal bacteria, *Bacteroides, Bifidobacterium, Lactobacillus, Clostridium leptum* group, and *C. coccoides* were quantitative analyzed by qPCR. As shown in Figure 6(E), there was no significant difference in the total level of intestinal bacteria among the four groups. However, some beneficial bacteria such as *Bidobacterium* (*p <* .01) and *Lactobacillus* (*p <* .001) species in mice were significantly reduced upon treatment with DSS. Treatment with CD–Cur–CANPs significantly reversed the decrease in *Bifidobacterium* (*p <* .05) and *Lactobacillus* (*p <* .001) levels caused by DSS treatment, while the effect of free CD–Cur treatment was not as pronounced as that of the CD–Cur–CANPs group. No significant difference was observed in the levels of *Bacteroides, C. leptum*, and *C. coccoides* among the four groups. The results indicated that CD–Cur–CANPs modified the relative abundance of gut microbial composition, some of which may play a central role in the alleviation of DSS-induced colitis.

## Discussion

Colon-targeted drug delivery offers an efficacious treatment for UC to avoid systemic absorption and potential side effects (Arévalo-Pérez et al., 2020). However, ODDS for colonic region presents certain challenges. Natural polymer-based nanocarriers have biocompatibility and biodegradability, which makes them suitable for physiological drug delivery approaches (Pushpamalar et al., 2021). In this study, a core–shell NPs were successfully prepared using CD–Cur as core and CANPs as shell. The strong electrostatic interaction of the shells leads to shrinkage and gel formation at low pH, thereby protecting the core from GIT and the aggressive gastric environment. The *in vitro* release indicated that only 12% and 27% of the Cur was released at pH values of the stomach (pH 1.2) and the upper part of the small intestine (pH 6.8), respectively (Figure 2(B)). These pH-dependent characteristics enable the formed CD–Cur–CMNP to avoid the initial burst of drug release in the upper GIT and ensure the subsequent sustained drug release in the colonic pH.

Recently, advanced stimuli-responsive controlled release systems have received increasing attention for drug delivery. These systems can regulate the release of entrapped drug molecules by specific external stimuli such as light, temperature, magnetic fields, or by internal enzymes (Ruiz-Hernández et al., 2011). New synthetic pH-dependent colon delivery systems combination with timed-release, ion-sensitive or ROS-response property are being developed (Dasgupta et al., 2018; Sacks et al., 2018). Enzyme as a kind of unique stimulant has been more and more used as trigger in controlled release system (Xiong et al., 2012). α-Amylase is a key enzymes involved in the carbohydrate digestion process (Dhital et al., 2017). Herein, the synthetic CD–Cur–CANPs is not only pH-dependent, but also drastic release in the presence of α-amylase. *In vitro* release curve showed that the released Cur from CD–Cur–CANPs was significantly higher in the presence of α-amylase (>90% within 4 h) compared to the absence of α-amylase (36.7%, 24 h). Triggered release of Cur in the presence of α-amylase is owing to the β-CD degradation resulting ring opening and chain scission in β-CD. These results indicated that the presence of α-amylase significantly promoted the release rate of Cur *in vitro*. Meanwhile, the actual effect of enzyme-induced release *in vivo* needs further verification.

Cur is recognized as a safe food additive by the US Food and Drug Administration (FDA) and has been studied as an anti-inflammatory drug for the treatment of UC. Nonetheless, its use is limited due to its low water solubility and bioavailability, poor intestinal absorption, and rapid systemic elimination (Anand et al., 2007). Specially, when Cur was used orally in clinical trials, weak therapeutic effects were observed because of inefficient drug delivery into the inflamed colon tissues. The hydrophobic cavity of CDs enables them to form inclusion complexes with a wide variety of poorly water-soluble and size matched guest molecules via the host–guest interactions. Herein, combination of the results obtained from *in vitro* cellular uptake of macrophages (Figure 4), *in vivo* biodistribution in GIT and accumulation in colonic tissue (Figure 3), our synthetic CD–Cur–CANPs indicated tremendous therapeutic potential in the treatment of mice colitis.

Gut microbiota is a complex and dynamic ecosystem that controls the permeability of the gastrointestinal mucosa and the host immune system, which is closely associated with UC (Ahlawat et al., 2021). However, the present ODDSs for the treatment of UC rarely consider the effects of intestinal homeostasis and gut microbiota. Our previous studies shown that chitosan oligosaccharides (COS) have effective modifying effect on gut microbiota (He et al., 2020). Meanwhile, our previous study also indicated that unsaturated alginate oligosaccharides (UAOS) with C4 and C5 double-bond, enzymatic degradation products of alginate, showed great effect on gut microbiota modulation (Li et al., 2020). In this study, low molecular weight chitosan and enzymatic unsaturated alginate were prepared in our lab and selected as polymer shells. Current studies have shown that Cur is therapeutically effective against UC and regulative effect on gut microbiota (Grammatikopoulou et al., 2018; Sadeghi et al., 2020; Shabbir et al., 2021). Quantitative analysis indicated that CD–Cur–CANPs significantly reversed the decrease in *Bifidobacteria* and *Lactobacilli* induced by DSS treatment. These results demonstrated that our prepared CD–Cur–CANPs may be a promising synergistic gut microbiota-targeting approach in UC treatment.

## Conclusions

In this study, a pH-sensitive and α-amylase-triggered controlled release system was successfully prepared using CD–Cur as core and CANPs as shell. Oral administration of CD–Cur–CANPs had an efficient therapeutic efficacy, strong colonic biodistribution and accumulation, rapid macrophage uptake, promoted colonic epithelial barrier integrity and modulated production of inflammatory cytokines, which reshaped the gut microbiota in mice with DSS-induced colitis. Overall, the colon-targeted oral delivery system developed in the current study has great potential for application in UC therapy.

## Supplementary Material

Supplemental Material
