# Supplementary material for: An efficient enzyme-triggered controlled release system for colon-targeted oral delivery to combat dextran sodium sulfate (DSS)-induced colitis in mice
Source: Drug Deliv. 2021 Jun 12;28(1):1120–31. doi: 10.1080/10717544.2021.1934189 (PMC8205034; doi:10.1080/10717544.2021.1934189)
Supplement: Supplemental Material [file IDRD_A_1934189_SM6469.doc]

**Supplymentary Materials**


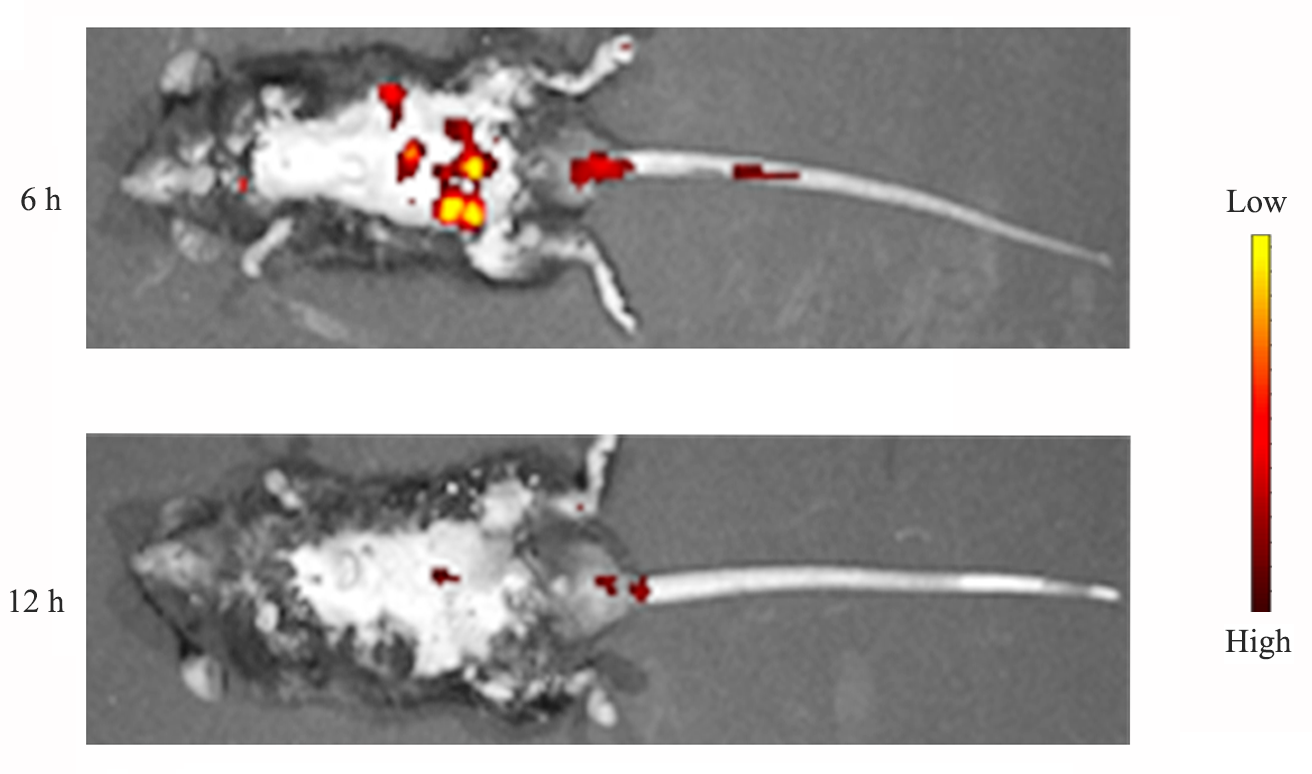


**Figure S1.** Determination of *in vivo* biodistribution of CD-Cur-CMNPs. The images were obtained using small animal *in vivo* 3D imaging system.


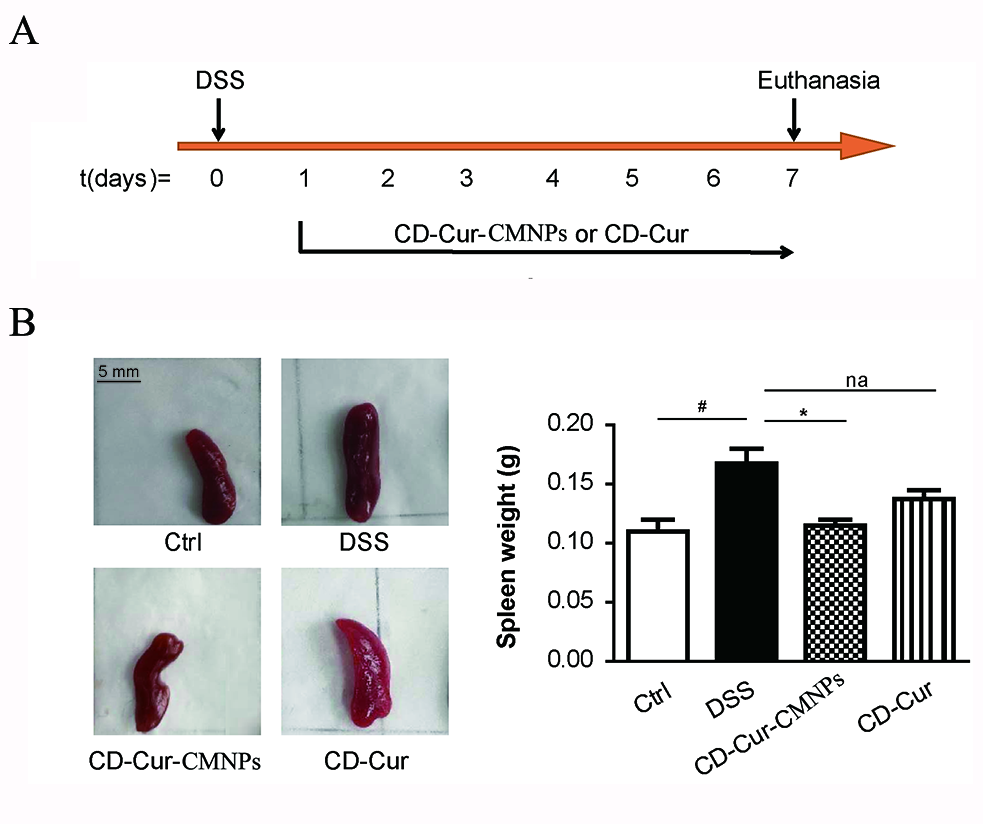


**Figure S2.** Scheme of DSS-induced colitis mice (A) and change in spleen weight of each group (B). Compared to control group, ^#^*P<*0.05, ^##^*P<*0.01, ^###^*P<*0.001. Compare to DSS group, ^*^*P<*0.05, ^**^*P<*0.01, ^***^*P<*0.001, na: no significance.

**Table S1. Evaluation of the DAI.**

| Score | Weight loss (%) | Stool consistency | Occult/gross bleeding | |
| --- | --- | --- | --- | --- |
| 0 | 0 | Normal | Normal |  |
| 1 | 1-5 |  |  |  |
| 2 | 5-10 | Loose stool | Hemoccult positive | |
| 3 | 10-15 |  |  |  |
| 4 | >15 | Diarrhea | Gross bleeding | |

**Table S2. The primers sequences used for PCR reaction (gene expression).**

| **Gene name** | **Primers sequences** |
| --- | --- |
| TNF-α | Forward: 5’-TAGCCAGGAGGGAGAACAGA-3’  Reverse: 5’-TTTTCTGGAGGGAGATGTGG-3’ |
| IL-1β | Forward: 5’-TTGAAGAAGAGCCCATCCTC-3’  Reverse: 5’-CAGCTCATATGGGTCCGAC-3’ |
| IL-6 | Forward: 5’-CCGGAGAGGAGACTTCAC-3’  Reverse: 5’-TCCACGATTTCCCAGAGA-3’ |

**Table S3. The primers sequences used for PCR reaction (microbiota composition).**

| **Microbial groups** | **Primers sequences** |
| --- | --- |
| All bacteria | Forward: 5’-ACTCCTACGGGAGGCAGCAG-3’  Reverse: 5’-ATTACCGCGGCTGCTGG-3’ |
| *Bacteroides* | Forward: 5’-GAGAGGAAGGTCCCCCAC-3’  Reverse: 5’-CGCTACTTGGCTGGTTCAG-3’ |
| *Bifidobacterium* | Forward: 5’ -CGCGTCYGGTGTGAAAG-3’  R Reverse: 5’-CCCCACATCCAGCATCCA-3’ |
| *Lactobacillus* | Forward: 5’-GAGGCAGCAGTAGGGAATCTTC-3’  Reverse: 5’-GGCCAGTTACTACCTCTATCCTTCTTC-3’ |
| *Clostridium leptum* group | Forward: 5’-GCACAAGCAGTGGAGT-3’  Reverse: 5’-CTTCCTCCGTTTTGTCAA-3’ |
| *Clostridium coccoides* | Forward: 5’-AAATGACGGTACCTGACTAA -3’  Reverse: 5’-CTTTGAGTTTCATTCTTGCGAA-3’ |
